# The incidence of pregnancy hypertension in India, Pakistan, Mozambique, and Nigeria: A prospective population-level analysis

**DOI:** 10.1371/journal.pmed.1002783

**Published:** 2019-04-12

**Authors:** Laura A. Magee, Sumedha Sharma, Hannah L. Nathan, Olalekan O. Adetoro, Mrutynjaya B. Bellad, Shivaprasad Goudar, Salécio E. Macuacua, Ashalata Mallapur, Rahat Qureshi, Esperança Sevene, John Sotunsa, Anifa Valá, Tang Lee, Beth A. Payne, Marianne Vidler, Andrew H. Shennan, Zulfiqar A. Bhutta, Peter von Dadelszen, the CLIP Study Group

**Affiliations:** 1 School of Life Course Sciences, Faculty of Life Sciences and Medicine, King’s College London, London, United Kingdom; 2 Department of Obstetrics and Gynaecology, University of British Columbia, Vancouver, British Columbia, Canada; 3 Olabisi Onabanjo University, Ago Iwoye, Ogun State, Nigeria; 4 Jawaharlal Nehru Medical College, KLE Academy of Higher Education and Research, Belagavi, Karnataka, India; 5 Centro de Investigação em Saúde da Manhiça, Manhiça, Mozambique; 6 S Nijalingappa Medical College, Hanagal Shree Kumareshwar Hospital and Research Centre, Bagalkote, Karnataka, India; 7 Centre of Excellence, Division of Woman and Child Health, Aga Khan University, Karachi, Pakistan; 8 Babcock University Teaching Hospital, Ilishan-Remo, Ogun State, Nigeria; 9 Centre for International Child Health, University of British Columbia, Vancouver, British Columbia, Canada; 10 Centre for Global Child Health, Hospital for Sick Children, Toronto, Ontario, Canada; University of Manchester, UNITED KINGDOM

## Abstract

**Background:**

Most pregnancy hypertension estimates in less-developed countries are from cross-sectional hospital surveys and are considered overestimates. We estimated population-based rates by standardised methods in 27 intervention clusters of the Community-Level Interventions for Pre-eclampsia (CLIP) cluster randomised trials.

**Methods and findings:**

CLIP-eligible pregnant women identified in their homes or local primary health centres (2013–2017). Included here are women who had delivered by trial end and received a visit from a community health worker trained to provide supplementary hypertension-oriented care, including standardised blood pressure (BP) measurement. Hypertension (BP ≥ 140/90 mm Hg) was defined as chronic (first detected at <20 weeks gestation) or gestational (≥20 weeks); pre-eclampsia was gestational hypertension plus proteinuria or a pre-eclampsia-defining complication. A multi-level regression model compared hypertension rates and types between countries (*p <* 0.05 considered significant). In 28,420 pregnancies studied, women were usually young (median age 23–28 years), parous (53.7%–77.3%), with singletons (≥97.5%), and enrolled at a median gestational age of 10.4 (India) to 25.9 weeks (Mozambique). Basic education varied (22.8% in Pakistan to 57.9% in India). Pregnancy hypertension incidence was lower in Pakistan (9.3%) than India (10.3%), Mozambique (10.9%), or Nigeria (10.2%) (*p* = 0.001). Most hypertension was diastolic only (46.4% in India, 72.7% in Pakistan, 61.3% in Mozambique, and 63.3% in Nigeria). At first presentation with elevated BP, gestational hypertension was most common diagnosis (particularly in Mozambique [8.4%] versus India [6.9%], Pakistan [6.5%], and Nigeria [7.1%]; *p <* 0.001), followed by pre-eclampsia (India [3.8%], Nigeria [3.0%], Pakistan [2.4%], and Mozambique [2.3%]; *p <* 0.001) and chronic hypertension (especially in Mozambique [2.5%] and Nigeria [2.8%], compared with India [1.2%] and Pakistan [1.5%]; *p <* 0.001). Inclusion of additional diagnoses of hypertension and related complications, from household surveys or facility record review (unavailable in Nigeria), revealed higher hypertension incidence: 14.0% in India, 11.6% in Pakistan, and 16.8% in Mozambique; eclampsia was rare (<0.5%).

**Conclusions:**

Pregnancy hypertension is common in less-developed settings. Most women in this study presented with gestational hypertension amenable to surveillance and timed delivery to improve outcomes.

**Trial registration:**

This study is a secondary analysis of a clinical trial - ClinicalTrials.gov registration number NCT01911494.

## Abstract 2

Laura Magee and colleagues present a survey of hypertension levels in pregnant women based on data from clinical trials in low and middle income countries.

## Author summary

**Why was this study done?:**

- High blood pressure in pregnancy is thought to occur in up to 1 in 10 pregnant women.
- Most information is from more-developed countries or from referral hospitals in less-developed countries, which is regarded to overestimate the occurrence of this condition.

**What did the researchers do and find?:**

- As part of the Community-Level Interventions for Pre-eclampsia (CLIP) trials in 27 geographical clusters (India, Pakistan, Mozambique, and Nigeria; 2013–2017), we undertook standardised blood pressure measurement in 28,420 pregnancies, in women’s homes or their primary health centres.
- Hypertension occurred in about 10% of pregnancies by standardised measurement, usually based on elevation of only the bottom (diastolic) number and often diagnosed for the first time postpartum; rates were 2%–7% higher when diagnoses from clinical care were included.
- At first presentation with elevated blood pressure, most (7%–8%) hypertension was pregnancy-induced (‘gestational’) without other associated problems.

**What do these findings mean?:**

- In our study countries, hypertension in pregnancy was more common than previously estimated.
- Most hypertension could only be measured with a blood pressure device, and not by using a cuff and palpation alone.
- Most women presented with gestational hypertension that is amenable to surveillance and timed delivery to decrease adverse outcomes for mothers and their offspring.
- Many women presented with hypertension only postpartum, highlighting the need for ongoing surveillance of maternal well-being after birth.

## Introduction

The United Nations Sustainable Development Goal (SDG) 3.1 aims to reduce the global maternal mortality ratio to less than 70 per 100,000 live births by 2030 [1]. The SDGs aim to maintain the momentum of the Millennium Development Goals, which catalysed a global reduction in maternal deaths from approximately 390,000 in 1990 to 275,000 in 2015 [1,2]. The burden of maternal mortality remains disproportionately borne by women in less-developed countries, particularly in sub-Saharan Africa (66%, 201,000 deaths) and southern Asia (22%, 66,000 deaths) [3].

One of the leading causes of maternal death (and disability) worldwide is pregnancy hypertension [4]. Incidence estimates from less-developed countries have varied from 4.0% to 12.3% [4–9]. These estimates are based on facility-based cross-sectional cohort studies, which are likely to overestimate rates compared with population-based data. Thus, the true incidence is unknown. Adding further to these measurement challenges are a lack of access to quality antenatal care and blood pressure (BP) measurement, the lack of a standardised definition for pre-eclampsia, and variable quality and coverage of routine health information systems; for example, national demographic health surveys have reported that only half of women have a BP measurement during antenatal care in Mozambique (54.4%) [10], and although the corresponding figure in India is 89%, only half of women report receiving at least 4 antenatal care visits [11].

Pregnancy hypertension is classified into 3 major categories: pre-existing (chronic) hypertension, gestational hypertension, and pre-eclampsia (which includes eclampsia). In the World Health Organization (WHO) Multicountry Survey on Maternal and Newborn Health—a cross-sectional hospital-based survey of 313,030 women admitted to 357 health facilities in 29 countries across Africa, Asia, Latin America, and the Middle East—0.29% of women were reported to have chronic hypertension (range 0.21% [Africa] to 0.32% [Western Pacific]) [12], 2.2% pre-eclampsia (excluding eclampsia; range 1.4% [Middle East] to 3.9% [Africa]), and 0.28% eclampsia (range 0.14% [Western Pacific] to 0.55% [Africa]) [12,13]; gestational hypertension was excluded from the WHO multicountry survey estimates. Other published rates have varied considerably; in hospital-based retrospective or prospective studies of variable size, gestational hypertension has been reported to complicate 2%–3% of deliveries in Karachi, Pakistan [14], 6.6% in south India [15], and 28.9% in southwest Nigeria [16,17].

We sought to establish reliable estimates of pregnancy hypertension incidence and type in 4 less-developed settings in southern Asia (India and Pakistan) and sub-Saharan Africa (Mozambique and Nigeria), using BP data gathered in the community using a validated semi-automated BP device from the Community-Level Interventions for Pre-eclampsia (CLIP) cluster randomised controlled trials (NCT01911494) [18].

## Methods

This is a secondary, planned analysis of data collected in the 4 countries and 27 intervention clusters of the CLIP cluster randomised controlled trials (NCT01911494) [18], in India (*N* = 6, Karnataka State), Pakistan (*N* = 10, Sindh Province), Mozambique (*N* = 6, Maputo and Gaza Provinces), and Nigeria (*N* = 5, Ogun State). A STROBE checklist is provided (S2 Table).

In brief, pregnant women (aged 15–49 years in India, Pakistan, and Nigeria, and 12–49 years in Mozambique) were enrolled in the CLIP trials when they first declared their pregnancy and following informed consent. The CLIP intervention consisted of community engagement and community health worker (CHW)–provided mobile health-guided clinical assessment, initial treatment, and referral to facility. Surveillance data were collected by a separate surveillance team, by quarterly household surveys in Pakistan, 6-monthly household surveys in Mozambique and Nigeria, and a research registry of facility records in India. The primary outcome was a composite of maternal, fetal, and newborn mortality and major morbidity. The protocol has been published [18] and is included S1 Text, along with the statistical analysis plan (S2 Text). The trials were approved by the University of British Columbia Research Ethics Board (H12-03497) and within each country (MDC/IECHSR/2013-14/A, India; 2590-Obs-ERC-13, Pakistan; 219/CNBS/13, Mozambique; OOUTH/DA.326/T/1/, Nigeria).

The CLIP intervention was implemented in primarily rural areas of India (February 2014 to October 2016), Pakistan (February 2014 to December 2016), Nigeria (March 2014 to January 2016), and Mozambique (February 2015 to February 2017). Within each country, a potential cluster consisted of an established unit of the health system (i.e., primary health centre [PHC] in India, union council in Pakistan, administrative post in Mozambique, and local government area in Nigeria), consisting of all relevant villages and PHCs (other than in India, where the PHC defined the cluster) within each unit. Potential clusters were chosen by the local team (based on similar healthcare infrastructure, accessibility to the surveillance team, and the absence of conflicting concurrent research activity). A random sample was then chosen, with restricted, stratified central randomisation (according to population size and region) to the intervention or to control (usual care).

In intervention clusters, CHWs were trained to provide mobile health-guided visits—supplementary pregnancy hypertension-oriented antenatal and postpartum care at home (India, Pakistan, and Mozambique) or at a PHC (Nigeria). Women in control clusters received usual care, advocated by WHO as BP measurement (using the device available) and proteinuria testing at each antenatal care visit at primary or other health centres; in these settings, most women receive 1 such visit at PHCs, and few women receive 4. In none of the study countries did CHWs usually either manage pregnancy hypertension or carry with them BP measurement equipment or antihypertensive medication; further details of their training and usual duties are detailed in S3 Text.

The CLIP intervention visits were guided by a novel mobile health application provided on Android tablets—Pre-eclampsia Integrated Estimate of Risk on the Move (POM)—that provided step-by-step guidance for clinical assessment (including oximetry in Pakistan and Mozambique), input of clinical data, and decision support for initial triage, transport, and treatment of women identified with pregnancy hypertension or emergency medical conditions [19]. This management was based on prediction of adverse maternal outcome and stillbirth, derived from incorporation into POM of the miniPIERS predictive model in hypertensive pregnancy [20]; this is a demographics-, symptom-, and sign-based model for use in low-resource settings to identify the risk of adverse maternal outcome among women with pregnancy hypertension. Usability and feasibility testing supported experimental implementation into clinical care [21].

POM-guided clinical assessment consisted of (i) a visual scan and enquiry for evidence of an emergency condition that would warrant immediate referral (i.e., unconsciousness, stroke or seizure, significant vaginal bleeding, or lack of fetal movement in the preceding 12 hours); (ii) signs or symptoms suggestive of end-organ involvement of pre-eclampsia (i.e., maternal symptoms of headache or visual disturbances, chest pain or dyspnoea, epigastric or right upper quadrant abdominal pain, or vaginal bleeding with abdominal pain); (iii) BP measurement; (iv) measurement of dipstick proteinuria both during the first antenatal visit and at all visits at which the woman was hypertensive; and (v) blood oxygen saturation assessment, using an Android mobile-phone- or tablet-adapted pulse oximeter to further improve risk stratification (Mozambique and Pakistan only) [22]. Clinical assessments were recommended every 4 weeks until 28 weeks gestation, every 2 weeks from 28 to 35 weeks, weekly from 36 weeks until delivery, once within 24 hours of birth, and postnatally around postpartum days 3, 7, and 14; in Nigeria, visits were opportunistic when women attended the PHC.

BP was measured using a semi-automated oscillometric device, validated for use in pregnancy and pre-eclampsia (Microlife 3AS1-2) [23]. CHWs were trained to have women rest for 5 minutes and then measure BP in a standardised fashion, at least twice (S3 Table). All BP readings were manually entered into POM, which averaged them as the BP for that visit; the first and second readings were averaged unless they were more than 10 mm Hg different, in which case a third reading was requested and the second and third readings were averaged [24]. The POM device provided guidance for community-initiated treatment (i.e., methyldopa 750 mg for systolic BP [sBP] ≥ 160 mm Hg and intramuscular magnesium sulphate 10 g for miniPIERS score > 25%) and referral (within 24 hours for sBP ≥ 140 mm Hg, and within 4 hours for emergency conditions, sBP ≥ 160 mm Hg, or miniPIERS score > 25%) [19].

Hypertension was defined as sBP ≥ 140 mm Hg or diastolic BP (dBP) ≥ 90 mm Hg. All normotensive POM-guided visits were included until the woman was found to be hypertensive, if applicable. Hypertension was defined as chronic (first detected at <20 weeks gestation) or gestational (first detected at ≥20 weeks) [25]. The rates for pre-eclampsia (and eclampsia) were estimated among pregnancies of women who were assessed and normotensive at <20 weeks gestation or who were first assessed at ≥20 weeks; pre-eclampsia was defined as gestational hypertension with proteinuria or 1 or more relevant end-organ complications [25]. Eclampsia was defined as seizure associated with pregnancy hypertension and was considered as a form of pre-eclampsia (detailed definitions in S4 Table).

The data entered on the POM devices from women in intervention clusters were synchronised and stored on Research Electronic Data Capture servers, and transferred regularly to the University of British Columbia CLIP Co-ordinating Centre in Vancouver.

Trial surveillance data (i.e., baseline characteristics, processes of care and delivery, and adverse maternal, perinatal, and neonatal outcomes) were based on maternal report and collected by an independent team of fieldworkers trained in household surveillance (Pakistan, Mozambique, and Nigeria) or were collected by review of facility records (India), initially on paper and then electronically using tablets. Surveillance occurred quarterly in Pakistan, 6-monthly in Mozambique, and prospectively in near real time via the Global Network for Women’s and Children’s Health Research’s Maternal Newborn Health Registry in India [26]. In Nigeria, trial surveillance data (which included extended baseline characteristics and outcomes) were not available as surveillance was suspended and the trial closed shortly after the pilot phase because of challenges identified in data entry from paper case report forms to the electronic database; of note, POM device data in Nigeria (and all CLIP countries) were entered directly into POM by the CHWs, a different team from those trained to conduct trial surveillance. Data management protocols ensured data security by encryption, data tracking through user identification numbers and audit trails, and effective data synchronisation between devices within the same study cluster and with the Research Electronic Data Capture server. For this analysis, we included pregnancies of women in intervention clusters who had received at least 1 POM-guided visit and who had delivered by trial end. We excluded pregnancies of women who were still on follow-up (i.e., undelivered) to avoid underestimation of hypertension incidence. Our focus was on the type of pregnancy hypertension at first presentation with elevated BP in the community, to provide data for clinicians to inform individual counselling and care; these data were derived from POM. Of additional interest was pregnancy hypertension incidence and type overall, to provide data for those responsible for planning resource allocation; these data were derived from both POM and trial surveillance that included information around the time of delivery.

The intervention clusters in each country were treated as 1 cohort for the purposes of our primary analysis comparing pregnancy hypertension epidemiology (i.e., incidence, type, and severity) between countries. In sensitivity analyses, hypertension incidence and type at presentation were evaluated (i) among all pregnancies of women who received POM-guided visits, regardless of delivery status (i.e., this analysis included women who were still undelivered at the end of the trial), (ii) following adjustment for baseline maternal characteristics (i.e., age, parity, and maternal basic education, except in Nigeria where data were not available), and (iii) following incorporation of trial surveillance data that included data and diagnoses from the household surveys and review of facility records. No adjustment was made for gestational age at first POM-guided care visit or total number of visits because the relationship between gestational age at a visit and probability of diagnosing hypertension is inconsistent, with a nadir of BP at approximately 20 weeks, and the predominance of hypertension at term gestational age [24]. Continuous data were summarised by mean and standard deviation or median and interquartile range, as appropriate, to avoid making assumptions about the distribution of the data. Categorical data were summarised by number and proportion. Pairwise country comparisons were made by chi-squared test and Wilcoxon signed rank test, as appropriate. Between-country comparisons were made by chi-squared test for categorical variables and Kruskal–Wallis test for continuous variables, as appropriate. Hypertension rates between countries were compared using logistic regression, adjusting for country. Additional explanatory analyses were undertaken to explore the basis of any between-country differences based on women’s baseline characteristics. Analyses were performed using R programming software (version 3.3.2). *p <* 0.05 was considered statistically significant.

## Results

**Fig 1:**
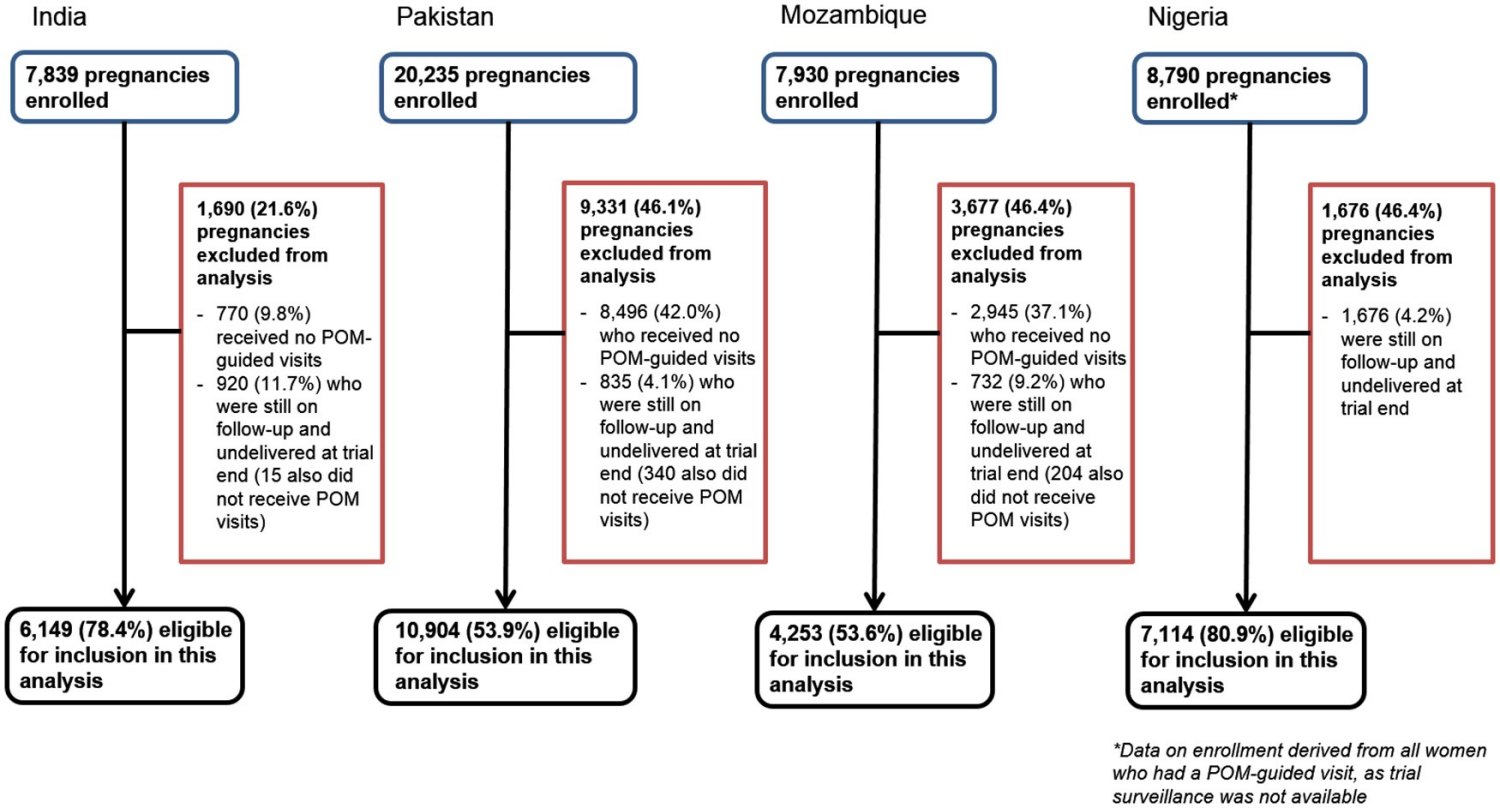
Pregnancies enrolled and included in the study. POM, Pre-eclampsia Integrated Estimate of Risk on the Move.

Of the 44,794 pregnancies in CLIP intervention clusters, 12,211 (27.2%) did not receive at least 1 POM-guided visit, and 4,163 (9.3%) were not delivered by trial end, leaving 28,420 (63.4%) pregnancies for inclusion in this analysis (Fig 1).

There were statistical differences between countries for all baseline pregnancy characteristics (Table 1). At enrolment in CLIP, women in India and Mozambique were younger than were those in Pakistan and Nigeria, but the absolute differences were very small. About one-third of women were nulliparous, except in Pakistan, where the proportion was closer to one-fifth. Women in India were enrolled earlier, usually at the end of the first trimester, compared with enrolment predominantly in the second trimester in other countries, particularly in Mozambique. Rates of maternal basic education were low overall, particularly in Pakistan. There were more multiple pregnancies in Mozambique, but the proportion was still small (<3%). CLIP data did not include information about smoking, body mass index, or prior pre-eclampsia. In all countries, women who received 1 or more POM-guided visits and those who did not were similar at baseline, although median gestational age at enrolment in CLIP was 2–4 weeks earlier for the former than for the latter (S5 Table); information was unavailable in Nigeria (see Methods).

**Table 1:** Patient characteristics for women who had received 1 or more POM-guided visits and were delivered by end of CLIP trial.

Characteristic	India (*N* = 6,149)	Pakistan (*N* = 10,904)	Mozambique (*N* = 4,253)	Nigeria (*N* = 7,114)	*p*-Value*
**Maternal age (years)**	23 [20, 25]	28 [25, 30]	23 [19, 30]	27 [23, 31]	<0.001
Missing	0	22 (0.2%)	146 (3.4%)	10 (0.1%)	
**Nulliparous**	2,230 (36.3%)	2,481 (22.75%)	1,285 (30.2%)	2,180 (30.6%)	<0.001
**Maternal basic education**^†^	3,560 (57.9%)	2,486 (22.8%)	2,486 (55.0%)	Unknown‡	<0.001
**Multiple pregnancies**	53 (0.9%)	86 (0.8%)	105 (2.5%)	Unknown‡	<0.001
**GA at CLIP enrolment (weeks)**	10.4 [7.9, 14.1]	18.7 [13.6, 24.6]	25.9 [19.5, 31.0]	16.6 [13.4, 18.4]	<0.001
**Pregnancies with antenatal visit(s)**	6,120 (99.5%)	10,885 (99.8%)	4,234 (99.6%)	7,004 (98.5%)	<0.001
GA at first POM-guided visit (weeks)	13.4 [9.5, 20.1]	21.9 [16.4, 28.4]	27.3 [22.4, 32.7]	28.0 [22.2, 33.4]	<0.001
GA uncertain (antenatal or postpartum) at first POM-guided visit	59 (0.96%)	50 (0.5%)	40 (0.9%)	164 (2.3%)	
First POM-guided visit at <20 weeks	4,545 (73.7%)	4,430 (40.6%)	638 (15.0%)	1,147 (16.1%)	<0.001
First POM-guided visit at ≥20 weeks	1,564 (25.4%)	6,425 (58.9%)	3,575 (84.1%)	5,827 (81.9%)	<0.001
GA uncertain at first antenatal POM-guided visit	40 (0.6%)	49 (0.4%)	40 (0.9%)	140 (2.0%)	
**Pregnancies with postpartum visit(s)**	3,759 (61.1%)	8,439 (77.4%)	3,434 (80.7%)	2,416 (34.0%)	<0.001
**GA at delivery (weeks)**	39.3 [37.4, 40.4]	38.7 [36.3, 40.7]	39.3 [37.4, 41.0]	39.3 [37.4, 40.7]	
**Visits per pregnancy**					
Number of antenatal visits per pregnancy	8.0 [3.0, 12.0]	3 [2.0, 5.0]	4.0 [2.0, 6.0]	2.0 [1.0, 4.0]	<0.001
Number postpartum visits per pregnancy	2.0 [1.0, 4.0]	2.0 [1.0, 3.0]	2.0 [1.0, 3.0]	3.0 [2.0, 4.0]	<0.001

Data are number (%) of pregnancies or median [IQR].

*The *p*-value was based on comparisons of all groups by Kruskal-Wallis test for continuous variables, and chi-square test for categorical variables, as appropriate.

^†^Maternal basic education is defined as ≥8 years of schooling (India), ≥5 years of schooling (Pakistan), or achievement of Grade 5 or above (Mozambique).

^‡^Trial surveillance data was not available for Nigeria.

CLIP, Community-Level Interventions for Pre-eclampsia; GA, gestational age; POM, Pre-eclampsia Integrated Estimate of Risk on the Move.

In ≥98.5% of informative pregnancies, there was an antenatal POM-guided visit, usually within a few weeks of enrolment, other than in Nigeria, where there was a 3-month delay on average (Table 1). As such, the first POM-guided visit occurred before 20 weeks gestation in most (73.4%) pregnancies in India, just under half (40.6%) in Pakistan, and a distinct minority (<20%) in Mozambique and Nigeria. In most pregnancies, there was a postpartum visit, other than in Nigeria. Given the median gestational age at delivery, the median number of POM-guided visits received by women was lower than the frequency recommended in the CLIP protocol, in India (i.e., 10 versus 14 recommended), Pakistan (i.e., 5 versus 11 recommended), and Mozambique (i.e., 6 versus 10 recommended); data were not available in Nigeria. At the majority of the 168,997 POM-guided visits in all countries, BP was measured, both antenatally and postpartum (Table 2). As per the CLIP protocol, proteinuria was measured at >90% of the first antenatal visits, and 91.2%–96.7% of subsequent antenatal visits at which the woman was hypertensive, in all but Nigeria, where the proportion was lower (80.7%).

**Table 2:** Quality and nature of CLIP visits with POM mobile health application in intervention clusters in CLIP.

Characteristic	India (*N* = 6,149)	Pakistan (*N* = 10,904)	Mozambique (*N* = 4,253)	Nigeria (*N* = 7,114)	*p*-Value*
**Total number of visits performed**	57,679	56,296	26,544	28,468	—
Antenatal	48,030 (83.3%)	38,386 (68.2%)	18,421 (69.4%)	21,510 (75.6%)	—
Postpartum	9,647 (16.7%)	17,909 (31.8%)	8,122 (30.6%)	6,956 (24.4%)	—
Timing uncertain	2 (0.0%)	1 (0.0%)	1 (0.0%)	2 (0.0%)	—
**Quality of visits**					
Number of visits at which BP measured	57,299 (99.3%)	56,234 (99.9%)	26,323 (99.2%)	28,264 (99.3%)	<0.001
Antenatal	47,694 (99.3%)	38,339 (99.9%)	18,278 (99.2%)	21,313 (99.1%)	
Postpartum	9,604 (99.6%)	17,895 (99.9%)	8,045 (99.1%)	6,950 (99.9%)	
Timing uncertain	1	0	0	1	
Proteinuria measured^†^					
At first antenatal POM-guided visit (of those with an antenatal visit)	5,676 (92.8%)	10,769 (98.9%)	4,143 (97.9%)	6,372 (91.0%)	<0.001
At subsequent antenatal POM-guided visit at which the woman was hypertensive	373 (91.2%)	235 (96.7%)	107 (94.7%)	175 (80.7%)	<0.001

Data are number (%) of women.

*The *p*-value was based on comparisons of all groups by chi-squared test (only relevant for comparison of quality of visits).

^†^The CLIP protocol specified that proteinuria should be measured at the first CLIP visit, and then at subsequent visits at which the woman was hypertensive. In Nigeria, proteinuria was measured at many subsequent visits regardless of BP status (12,796/21,354, 59.9%).

BP, blood pressure; CLIP, Community-Level Interventions for Pre-eclampsia; POM, Pre-eclampsia Integrated Estimate of Risk on the Move.

Approximately 10% of pregnancies in each of the 4 countries were identified as hypertensive at a POM-guided visit (Table 3). The incidence was highest in Mozambique and lowest in Pakistan. In most pregnancies, diagnostic criteria for hypertension were met based on isolated dBP ≥ 90 mm Hg, except in India, where the proportion was just under half. Few pregnancies (<10% in all but Mozambique) were hypertensive based only on isolated systolic hypertension. In few pregnancies overall was isolated diastolic hypertension later associated with systolic hypertension (i.e., 75/295 [29.8%] in India, 58/734 [10.3%] in Pakistan, 20/285 [8.6%] in Mozambique, and 34/457 [10.6%] in Nigeria, *p <* 0.001), other than in India, where women presented earlier and had significantly more BP assessments. At least 85% of identified hypertension was non-severe, least commonly in Nigeria.

**Table 3:** Diagnosis of hypertension and its timing among pregnancies of women at a POM-guided visit.

	India (*N* = 6,149)	Pakistan (*N* = 10,904)	Mozambique (*N* = 4,253)	Nigeria (*N* = 7,114)	*p*-Value*
**Hypertension**	636 (10.3%)	1,010 (9.3%)	465 (10.9%)	722 (10.2%)	0.009
**Diagnostic criteria**					
Both sBP ≥ 140 mm Hg and dBP ≥ 90 mm Hg	295 (4.8%)	219 (2.0%)	94 (2.2%)	195 (2.7%)	<0.001
Only sBP ≥ 140 mm Hg	46 (0.7%)	57 (0.5%)	86 (2.0%)	70 (1.0%)	<0.001
Only dBP ≥ 90 mm Hg	295 (4.8%)	734 (6.7%)	285 (6.7%)	457 (6.4%)	<0.001
**Severity**					
Non-severe (sBP 140–159 or dBP 90–109 mm Hg)	593 (9.6%)	946 (8.7%)	430 (10.1%)	625 (8.8%)	<0.001
Severe (sBP ≥ 160 or dBP ≥ 110 mm Hg)	43 (0.7%)	64 (0.6%)	35 (0.8%)	97 (1.4%)	<0.001
**Timing relative to delivery**					
Antenatal	512 (8.3%)	552 (5.1%)	290 (6.8%)	449 (6.3%)	<0.001
GA at diagnosis (weeks)	36.0 [32.0, 39.0]	33.0 [26.0, 36.0]	35.0 [30.0, 38.0)	35.0 [27.0, 38.0)	<0.001
GA uncertain	7/512 (1.4%)	3/552 (0.5%)	3/290 (1.0%)	15/449 (3.3%)	
Postpartum	124 (2.0%)	458 (4.2%)	175 (4.1%)	272 (3.8%)	<0.001
Timing (days postpartum)	10.0 [6.0, 15.0]	6.0 [3.0, 11.0]	7.0 [4.0, 13.0]	3.0 [1.0, 7.0]	<0.001
Timing uncertain	1 (0.02%)	0	0	0	
Had an antenatal POM-guided visit	124/124 (100.0%)	457/458 (99.8%)	173/175 (98.9%)	253/272 (93.0%)	
Timing of last POM-guided visit before delivery (days)	5.0 [3.0, 8.0]	22.0 [13.0, 31.0]	7.0 [4.0, 14.0]	12.0 [5.0, 25.0]	
Timing uncertain	0	0	0	1 (0.01%)	
**Hypertension type at first diagnosis**					
**Chronic hypertension**^†^	54/4,545 (1.2%)	65/4,430 (1.5%)	16/638 (2.5%)	32/1,147 (2.8%)	<0.001
**Gestational hypertension**^†^	372/5,416 (6.9%)	690/10,543 (6.5%)	349/4,156 (8.4%)	475/6,676 (7.1%)	
Diagnosed antenatally	273/5,416 (5.0%)	313/10,543 (3.0%)	203/4,156 (4.9%)	226/6,676 (3.4%)	
Diagnosed postpartum	99/5,416 (1.8%)	377/10,543 (3.6%)	146/4,156 (3.5%)	249/6,676 (3.7%)	
**Pre-eclampsia**^†^ **(including eclampsia)**	204/5,416 (3.8%)	251/10,543 (2.4%)	96/4,156 (2.3%)	199/6,676 (3.0%)	
Pre-eclampsia diagnostic criteria met^‡^					
Proteinuria	59/5,416 (1.1%)	88/10,543 (0.8%)	8/4,156 (0.2%)	72/6,676 (1.1%)	
Maternal symptoms	152/5,416 (2.8%)	224/10,543 (2.1%)	72/4,156 (1.7%)	146/6,676 (2.2%)	
Maternal signs (including eclampsia)	42/5,416 (0.8%)	62/10,543 (0.6%)	30/4,156 (0.7%)	87/6,676 (1.3%)	
No fetal movement	4/5,416 (0.1%)	6/10,543 (0.1%)	3/4,156 (0.1%)	1/6,676 (0.01%)	
GA at pre-eclampsia onset (weeks)	37.9 [35.5, 39.4]	35.6 [31.3, 38.2]	36.9 [31.5, 39.8]	36.0 [32.0, 39.3]	<0.001
GA < 34 weeks	36/204 (17.7%)	99/251 (39.4%)	33/96 (34.4%)	70/199 (35.9%)	<0.001
GA ≥ 34 weeks	168/204 (82.4%)	152/251 (60.6%)	63/96 (65.6%)	129/199 (64.8%)	<0.001
**Hypertension type not known**^ǁ^	6 (0.1%)	4 (0.04%)	4 (0.1%)	16 (0.2%)	0.004

Data are number (%) of women or median [IQR].

*The *p*-value was based on comparisons of all groups by Kruskal–Wallis test for continuous variables, and chi-squared test for categorical variables, as appropriate.

^†^Chronic hypertension incidence was estimated only among women who were assessed at <20 weeks gestation. Gestational hypertension and pre-eclampsia incidence were estimated among women who were previously assessed as normotensive at <20 weeks or assessed for the first time at ≥20 weeks. For complete definitions please see S3 Table.

^‡^Not mutually exclusive.

^‖^Unknown because GA at hypertension diagnosis not known.

dBP, diastolic blood pressure; GA, gestational age; sBP, systolic blood pressure; POM, Pre-eclampsia Integrated Estimate of Risk on the Move.

In most pregnancies, hypertension was diagnosed antenatally (particularly in India), in the mid-late third trimester (Table 3). However, in a substantial proportion of pregnancies (approximately 40%), hypertension was first diagnosed by a POM-guided visit postpartum. This occurred despite virtually all such women having been confirmed to be normotensive antenatally, a median of under 2 weeks before delivery in all but Pakistan, where the last antenatal POM-guided visit was a median of 3 weeks before delivery. Postpartum hypertension was usually diagnosed within 7 days of delivery, except in India; there, the incidence of postpartum hypertension was lowest, and the timing of diagnosis was the most remote from delivery (i.e., median 10 days postpartum).

The incidence of chronic hypertension, reported only in pregnancies with an antenatal POM-guided visit at <20 weeks gestation, was lower in India (1.2%) and Pakistan (1.5%) compared with Mozambique (2.5%) and Nigeria (2.8%) (Table 3). When hypertension appeared at ≥20 weeks gestation, gestational hypertension was most common, with a slightly higher rate in Mozambique (8.4%) than in the other countries (approximately 7%). Most hypertension was diagnosed antenatally in India and Mozambique, whereas about half was so diagnosed in Pakistan and Nigeria.

Pre-eclampsia incidence was similar in each of the 4 countries (2.3%–3.8% of women). Most cases were diagnosed based on a broad definition that did not mandate the presence of new proteinuria (see S3 Table for definitions). Approximately one-third of pregnancies with pre-eclampsia demonstrated new proteinuria (other than in Mozambique, where the rate was <10%), most women had relevant maternal symptoms, many had maternal signs (particularly in Nigeria), and few (<5%) had reduced fetal movement. Rarely, women first presented with eclampsia as their hypertensive disorder (1 case in India and 2 in Nigeria). Most pre-eclampsia was diagnosed antenatally and near term, with about two-thirds presenting at ≥34 weeks gestation; the exception was in India, where pre-eclampsia presented at a median of about 38 weeks. In the sensitivity analysis that adjusted for baseline maternal characteristics available, rates of hypertension by type remained different between countries (S6 Table).

In sensitivity analyses, inclusion of women who were undelivered at the end of the trial resulted in lower incidence estimates of hypertension in each country; this was true for any hypertension and for each type (S6 Table). Adjustment for maternal characteristics (age, parity, and education) revealed some differences of the 3 other countries from the comparator India (S7 Table): In Pakistan, hypertension incidence overall remained lower than in India (adjusted odds ratio [aOR] 0.75, 95% CI 0.67, 0.85; *p <* 0.001), and the observed lower rates of gestational hypertension (aOR 0.84, 95% CI 0.73, 0.97; *p* = 0.016) and pre-eclampsia (aOR 0.50, 95% CI 0.41, 0.63; *p <* 0.001) reached statistical significance. In Mozambique, results were similar to those from unadjusted analyses (not significant), except for a lower incidence of pre-eclampsia than in India (aOR 0.51, 95% CI 0.40, 0.66; *p <* 0.001). In Nigeria, adjustment for maternal age and parity (as education was unavailable) revealed a lower incidence of hypertension overall than in India (aOR 0.82, 95% CI 0.73, 0.92; *p <* 0.001), associated with a higher incidence of chronic hypertension (aOR 1.68, 95% CI 1.05, 2.70; *p* = 0.030) and a lower incidence of pre-eclampsia (aOR 0.52, 95% CI 0.40, 0.67; *p <* 0.001). Finally, when we included hypertension and relevant end-organ complications documented by trial surveillance until 6 weeks postpartum (except in Nigeria, where these data were unavailable), estimates of pregnancy hypertension incidence rose, and were 36% higher in India (14.0% versus 10.3%), 25% higher in Pakistan (11.6% versus 9.3%), and 54% higher in Mozambique (16.8% versus 10.9%) (Table 4). Also, the relative proportions of types of hypertension differed because this approach also accounted for progression to pre-eclampsia/eclampsia at either subsequent POM-guided visits or according to trial surveillance. Overall, chronic hypertension was rare (<1.0% of pregnancies in all countries), gestational hypertension most common (6%–12% of pregnancies, highest in Mozambique), and pre-eclampsia intermediate in incidence (3%–6%, highest in India).

**Table 4:** Diagnosis of hypertension based on POM-guided community care and trial surveillance*.

Hypertension type	India (*N* = 6,149)	Pakistan (*N* = 10,904)	Mozambique (*N* = 4,253)	Nigeria (*N* = 7,114)	*p*-Value^†^
Chronic hypertension (without pre-eclampsia)	39 (0.6%)	40 (0.4%)	14 (0.3%)	30 (0.4%)	0.047
Gestational hypertension (without pre-eclampsia)	464 (7.5%)	753 (6.9%)	497 (11.7%)	441 (6.2%)	<0.001
Pre-eclampsia (including eclampsia)	353 (5.7%)	468 (4.3%)	198 (4.7%)	235 (3.3%)	<0.001
From chronic hypertension	15/54 (27.8%)	25/65 (38.5%)	2/16 (12.5%)	2/32 (6.3%)	
From gestational hypertension	74/372 (19.9%)	129/690 (18.7%)	56/349 (16.1%)	34/475 (7.2%)	
Eclampsia	17 (0.3%)	13 (0.1%)	15 (0.4%)	3 (0.0%)	<0.001
Hypertension type uncertain	7 (0.1%)	4 (0.04%)	4 (0.1%)	16 (0.2%)	
**Overall estimate of pregnancy hypertension**	863 (14.0%)	1,265 (11.6%)	713 (16.8%)	722 (10.2%)	<0.001

Data are number (%) of pregnancies.

*Trial surveillance data were not available for Nigeria.

^†^The *p*-value was based on comparisons of all groups by chi-squared test for categorical variables.

POM, Pre-eclampsia Integrated Estimate of Risk on the Move.

## Discussion

In almost 30,000 pregnancies from 27 CLIP intervention clusters in sub-Saharan Africa and southern Asia, use of standardised BP measurement revealed an incidence of pregnancy hypertension of approximately 10%. The rate was slightly lower in Pakistan, but the difference was not explained by between-country differences in measurable baseline maternal and pregnancy characteristics.

Our community-based incidence estimates of pregnancy hypertension types revealed that chronic hypertension was least common (approximately 1%–3% of pregnancies), gestational hypertension most common (approximately 6%–8%), and pre-eclampsia intermediate in incidence (2%–4%); eclampsia was rare (<1%) and was included within estimates of pre-eclampsia. Most pre-eclampsia diagnoses were based on a broad definition rather than proteinuria alone (present in up to 33% of pre-eclampsia pregnancies).

Most of the hypertension detected was solely diastolic, particularly when non-severe; this means that ascertainment of BP by palpation, which detects only systolic hypertension, would be both inadequate and inaccurate for clinical care in these settings. Most hypertension was diagnosed antenatally, usually in the mid-third trimester, supporting WHO recommendations for increased frequency of antenatal care visits and BP measurement approaching term gestation [27]. Importantly, a substantial number of women were first diagnosed with hypertension postpartum (despite being normotensive at antenatal CHW visits), emphasising the importance of post-delivery BP measurements in clinical care beyond the WHO recommendation to measure BP within the 24 hours after delivery [28].

The major strengths of our study are the population-based nature of recruitment, and standardised BP measurements, using a pregnancy-validated BP device [23]. We had a large sample size and evaluated women in 4 less-developed sub-Saharan and southern Asian countries. We estimated rates of chronic hypertension only among women who presented at <20 weeks gestation, an important consideration because most women in all but India first present for antenatal care beyond the first half of pregnancy, when chronic hypertension can be diagnosed. We evaluated women using repeated community BP measurements in the days after birth (which are often not performed even in well-resourced settings).

Limitations of this analysis include the fact that we had BP measurements by standardised methods only from community care, as facility care was not the focus of the CLIP trial. Our trial surveillance included documentation of hypertension and related complications from facility records and from women themselves; these data were included in a sensitivity analysis that suggested that our community estimates are conservative, possibly related to substantial numbers of women presenting with hypertension at the end of their pregnancy or around labour and delivery, at which point care did not include CHW-led community visits. CHWs were unable to perform their community assessments as frequently as specified in the CLIP protocol, particularly the weekly visits from 36 weeks until delivery; as such, we may have missed the onset of hypertension just before delivery, as suggested by the higher incidence of hypertension when we incorporated non-standardised clinical assessments of BP from trial surveillance. We were unable to adjust for gestational age at the initiation of POM-guided visits or for the number of visits, given the lack of a consistent relationship between gestational age at a visit and the probability of diagnosing hypertension; however, we will explore the relationship between the number of POM-guided visits and outcomes in future planned ‘dose–response’ analyses (S2 Text). We applied internationally agreed-upon definitions of pregnancy hypertension type based on gestational age at presentation [25]; we recognise that a limitation of this approach is that women who were assessed for the first time at ≥20 weeks gestation and found to be hypertensive could have been labelled as having gestational hypertension when in fact they had chronic hypertension, with/without a secondary cause such as renal disease. However, our approach is recognised to be the relevant clinical approach given the propensity of pre-eclampsia to progress, which must, as much as possible, be under observation. We were unable to include all end-organ complications of pre-eclampsia, assessed clinically or through laboratory tests; however, trial surveillance did incorporate those complications that would have been diagnosed and treated at facility. We had only basic maternal characteristics available for use in our adjusted analysis of hypertension rates and types by country. No information was available on past history of chronic hypertension, so our rates for chronic hypertension may be underestimated, particularly as many women first presented for antenatal care in the second half of pregnancy and so chronic hypertension could not be assessed directly. Of course, an estimate from any region cannot be assumed to be automatically generalisable to the whole country.

To our knowledge, this is the first study of pregnancy hypertension incidence and type in less-developed countries that is both population-based in terms of participants and standardised in terms of BP measurement, by positioning and use of a pregnancy-validated BP device [23]. Prior estimates from less-developed countries have varied from 4.0% to 12.3% and come primarily from facility-based cross-sectional cohort studies [4–9]. While such studies have been thought to overestimate pregnancy hypertension incidence, our data suggest that this may not be true. Our estimates are at least as high as those from more-developed settings [4] and still probably conservative given the impact of the addition of non-standardised clinical BP assessments made around the time of delivery and documented by trial surveillance.

Our estimate of chronic hypertension incidence resembles the 1%–2% from more-developed settings [4]. Our data are unique from less-developed settings as many women were evaluated at <20 weeks gestation.

As in well-resourced settings, gestational hypertension was the most common type of pregnancy hypertension in our study population (approximately 6%–8%). The rates we found are as high or higher than those published from facility-based retrospective or prospective cohort studies: 6.9% in India (compared with 6.6% published [15]), 6.5% in Pakistan (versus 1.7% published [14]), and 7.1% in Nigeria (compared with highly variable estimates from 1.3% [17] to 28.9% [16]); we identified no comparable published data in Mozambique. Of note, the median gestational age at enrolment in Mozambique and Nigeria was over 20 weeks; although it is possible that some cases of gestational hypertension were actually cases of chronic hypertension, the incidence of gestational hypertension in Mozambique and Nigeria was similar to that in India, where the median gestational age of enrolment was 13 weeks.

The incidence of pre-eclampsia in our study was similar to published rates of 2%–4% in more-developed countries [4], but slightly higher than rates previously documented in our study countries: 3.8% in India (versus 2.0% published [12]), 2.4% in Pakistan (versus 1.2% published [12]), and 3.0% in Nigeria (versus 2.3%–4.2% [12,17,29]); there were no comparable published data in Mozambique. Few women presented with eclampsia during community-based visits. The incidence of pre-eclampsia was lower than that of gestational hypertension, despite the broad definition of pre-eclampsia used in the study (i.e., without mandatory proteinuria).

A novel finding of this study is the high proportion of women diagnosed with hypertension based on dBP alone, and the fact that most did not go on to develop systolic hypertension at subsequent POM-guided visits. While the pregnancy hypertension literature to date has focused on dBP as a diagnostic criterion [30], this practice has been based on the greater susceptibility of sBP to environmental influences, rather than a regard for dBP being more important. However, outside pregnancy, high isolated dBP is characteristic of hypertension in the young [31] (as in our population), among whom it is the measure associated with elevated cardiovascular risk; it is in older populations that this association is assumed by systolic hypertension [31].

Finally, we have documented high rates of new postpartum hypertension (approximately 20%–45% of all cases of hypertension) based on a median of 2 postpartum CHW-led community visits and normal BP at antenatal visits. Comparable rates in either less- or more-developed countries are unknown, but in the latter, a rate of 2% has been estimated during the period prior to hospital discharge [32,33].

CHWs can measure BP and respond to hypertension; this has implications beyond maternity care to the broader SDG agenda related to non-communicable diseases [1]. Pregnancy hypertension is at least as common in less-developed countries as it is in more-developed. During active community surveillance of antenatal BP, most women present with non-severe elevations of BP due to gestational hypertension. As such, the severity and type of hypertension are amendable to intervention, by antihypertensive therapy [34] or timed delivery to optimise outcomes, particularly where there is limited capacity for the healthcare infrastructure to respond to obstetric emergencies. Future research should address, in particular, postnatal BP measurement and management.

## Supporting information

S1 Table CLIP study group. (DOCX)

S2 Table STROBE checklist. (DOC)

S3 Table BP measurement protocol for CHWs. (DOCX)

S4 Table Definitions of pregnancy hypertension types. (DOCX)

S5 Table Baseline characteristics of women who received 1 or more POM-guided visits (versus those who did not). (DOCX)

S6 Table Sensitivity analysis including all women who had a POM-guided visit regardless of follow-up status. (DOCX)

S7 Table Sensitivity analysis including adjustment for baseline characteristics. (DOCX)

S1 Text CLIP protocol. (PDF)

S2 Text CLIP statistical analysis plan. (PDF)

S3 Text CHWs—current scope of practice. (DOCX)

## Abbreviations

aOR: adjusted odds ratio
BP: blood pressure
CHW: community health worker
CLIP: Community-Level Interventions for Pre-eclampsia
dBP: diastolic blood pressure
GA: gestational age
miniPIERS: Pre-eclampsia Integrated Estimate of Risk
PHC: primary health centre
POM: Pre-eclampsia Integrated Estimate of Risk on the Move
sBP: systolic blood pressure
SDG: Sustainable Development Goal
WHO: World Health Organization
