# Supplementary material for: The incidence of pregnancy hypertension in India, Pakistan, Mozambique, and Nigeria: A prospective population-level analysis
Source: PLoS Med. 2019 Apr 12;16(4):e1002783. doi: 10.1371/journal.pmed.1002783 (PMC6461222; doi:10.1371/journal.pmed.1002783)
Supplement: S1 Table — (DOCX) [file pmed.1002783.s001.docx]

**Table S1: CLIP Study Group**

| **Country** |  |
| --- | --- |
| CLIP Nigeria Working Group | Olalekan O. Adetoro, John O. Sotunsa, Akinmade A. Adepoju; Adebayo A. Akadri, Yusuf A. Adefabi Bola A. Idowu-Ajiboye, David A. Akeju, Olukayode A. Dada, Busola Ibiezugbe, John Imaralu, Ebunolwa Jaiyesimi, Chimaobi C. Nwankpa, Oluwafayokemi O Odubena, Ayodeji Oluwole, Bisi Orenuga, Adebimpe M. Osiberu, Abiodun Owoseje, Adekunle T. Solarin |
| CLIP Mozambique Working Group | Esperança Sevene, Eusébio Macete, Khátia Munguambe, Charfudin Sacoor, Anifa Vala, Helena Boene, Felizarda Amose, Rosa Pires, Zefanias Nhamirre, Marta Macamo, Rogério Chiaú, Analisa Matavele, Faustino Vilanculo, Ariel Nhancolo, Silvestre Cutana, Ernesto Mandlate, Salésio Macuacua, Cassimo Bique, Sibone Mocumbi, Emília Gonçálves, Sónia Maculuve, Ana Ilda Biz, Dulce Mulungo, Orvalho Augusto, Paulo Filimone, Vivalde Nobela, Corsino Tchavana, Cláudio Nkumbula |
| CLIP Pakistan Working Group | Rahat Qureshi, Zulfiqar A Bhutta, Zahra Hoodbhoy, Farrukh Raza, Sana Sheikh, Javed Memon, Imran Ahmed, Amjad Hussain |
| CLIP India Working Group | Mrutunjaya B Bellad, Umesh S Charantimath, Shivaprasad S Goudar, Geetanjali M Katageri, Avinash J Kavi, Amit P Revankar, Ashalata A Mallapur, Umesh Y Ramdurg, Shashidhar G Bannale, Vaibhav B Dhamanekar, Geetanjali I Mungarwadi, Narayan V Honnungar, Bhalachandra S Kodkany, Anjali M Joshi, Uday S Kudachi, Sphoorthi S Mastiholi, Chandrappa C Karadiguddi, Gudadayya S Kengapur, Namdev A Kamble, Keval S Chougala |
| CLIP UBC Working Group | Peter von Dadelszen, Laura A. Magee, Jeffrey Bone, Dustin Dunsmuir, Sharla K Drebit, Chirag Kariya, Tang Lee, Jing Li, Mansun Lui, Beth A. Payne, Asif R Khowaja, Diane Sawchuck, Sumedha Sharma, Domena K. Tu, Marianne Vidler, Ugochi V. Ukah, Mai-Lei Woo |

*CLIP (Community-Level Interventions in Pre-eclampsia)*
