# Supplementary material for: The incidence of pregnancy hypertension in India, Pakistan, Mozambique, and Nigeria: A prospective population-level analysis
Source: PLoS Med. 2019 Apr 12;16(4):e1002783. doi: 10.1371/journal.pmed.1002783 (PMC6461222; doi:10.1371/journal.pmed.1002783)
Supplement: S3 Table — (DOCX) [file pmed.1002783.s003.docx]

**Table S3: BP measurement protocol for community health workers**

| **Step** | Instruction |
| --- | --- |
| 1 | Let the woman rest (seated, no talking) for at least 5min before taking the first measurement. |
| 2 | Place cuff on either arm supported at heart level – on a table (or arm rest of the chair) with woman sitting with back against a chair, no tight clothing around upper arm, both feet on the floor, cuff 1-2cm above elbow. NOTE: Women must remain still (i.e. no movement or talking) while the measurement is being taken for an accurate reading. |
| 3 | Turn on machine* and inflate cuff by hand, the cuff will then deflate automatically. If the cuff has not been inflated to the correct pressure, the device will indicate this with a 'beeping' sound - inflate the cuff to 30mmHg higher than the previous inflation pressure. Try to keep the device as still as possible during cuff deflation or alternatively let it rest on the table during deflation. NOTE: if the result is an ‘error’ readout, repeat the above process. |
| 4 | Enter measurement into POM device. |
| 5 | Wait one minute (during which the woman should remain still with no movement or talking) and repeat BP measurement. All women will receive two BP measurements; an average of the two readings will be taken. NOTE: if the result is an error readout, repeat the above process |
| *All women require two BP measurements. If these are within 10mm of each other, POM will calculate the average of those measurements and this will be the BP for that visit. If however, the second measurement differs significantly (>10mmHg) from the first, a third measurement is required.* | |
| 6 | Take a third BP measurement, as before, if instructed to do so by POM. In this case, the second and third measurements will be averaged to determine BP for that visit. NOTE: if the third reading yields an ‘error’ readout, repeat the measurement. |

*BP (blood pressure), POM (PIERS-On-the-Move)*

** CRADLE BP device*
