# Supplementary material for: The incidence of pregnancy hypertension in India, Pakistan, Mozambique, and Nigeria: A prospective population-level analysis
Source: PLoS Med. 2019 Apr 12;16(4):e1002783. doi: 10.1371/journal.pmed.1002783 (PMC6461222; doi:10.1371/journal.pmed.1002783)
Supplement: S4 Table — (DOCX) [file pmed.1002783.s004.docx]

**Table S4: Definitions of pregnancy hypertension types**

| **Type** | **Definition** |
| --- | --- |
| **Chronic hypertension** | An average systolic BP≥140mmHg and/or a diastolic BP≥90mmHg first detected at <20 weeks’ gestation. |
| **Gestational hypertension** | An average systolic BP≥140mmHg and/or a diastolic BP≥90mmHg first detected at ≥20 weeks’ gestation. |
| **Pre-eclampsia** |  |
| At presentation with hypertension | Gestational hypertension with ≥1+ proteinuria or one/more end-organ complications of pre-eclampsia, all of which were diagnosed in the community by CLIP-trained community health care workers:  • maternal symptom (i.e., headache, visual symptoms, chest pain or dyspnoea, epigastric or right upper quadrant abdominal pain),  • maternal sign (severe hypertension, vaginal bleeding, decreased level of consciousness associated with severe hypertension, or stroke), or  • fetal manifestation (i.e., no fetal movement in the last 12 hours) |
| Progression to pre-eclampsia | Among women with chronic or gestational hypertension, development of proteinuria or an end-organ complication that defines pre-eclampsia (as above), as documented at a subsequent POM visit or by trial surveillance (i.e., seizure, stroke, coma, antepartum haemorrhage, DIC, dialysis, mechanical ventilation, blood transfusion, or stillbirth) |
| Final diagnosis after delivery and postnatal care | Pre-eclampsia at presentation with hypertension, progression to pre-eclampsia from chronic or gestational hypertension, or hypertension reported in trial surveillance with one/more end-organ complications of pre-eclampsia |
| **Eclampsia** | Gestational hypertension associated with seizure as diagnosed in the community by the CLIP-trained community health care worker. |
| Progression to eclampsia | Among women with chronic or gestational hypertension, development of seizure documented at a subsequent POM visit or by trial surveillance. |
| Final diagnosis after delivery and postnatal care | Eclampsia at presentation with hypertension, progression to eclampsia from chronic or gestational hypertension, or hypertension and seizure reported in trial surveillance. |

*CLIP (Community-Level Interventions in Pre-eclampsia), DIC (disseminated intravascular coagulation), POM (PIERS-On-the-Move)*
