# Supplementary material for: The incidence of pregnancy hypertension in India, Pakistan, Mozambique, and Nigeria: A prospective population-level analysis
Source: PLoS Med. 2019 Apr 12;16(4):e1002783. doi: 10.1371/journal.pmed.1002783 (PMC6461222; doi:10.1371/journal.pmed.1002783)
Supplement: S5 Table — (DOCX) [file pmed.1002783.s005.docx]

**Table S5: Baseline characteristics of women who received POM-guided care (vs. those who did not) ***

|  | **India** | | | **Pakistan** | | | **Mozambique** | | |
| --- | --- | --- | --- | --- | --- | --- | --- | --- | --- |
|  | **Received POM (N=6149)** | **Did NOT receive POM (N=770)** | **p** | **Received POM (N=10,904)** | **Did NOT receive POM (N=8496)** | **P** | **Received POM (N=4253)** | **Did NOT receive POM (N=2945)** | **p** |
| **Maternal age** | 23.0  [20.0; 25.00] | 23.0  [20.0; 25.0] | 0.76 | 28.0  [25.0; 30.0] | 28.0  [25.0; 30.0] | 0.26 | 23.0  [19.0; 30.0] | 24.0  [19.0; 31.0] | 0.72 |
| *Missing* | 0 | 0 |  | 22 (0.20%) | 10 (0.1%) |  | 0 | 0 |  |
| **Nulliparous** | 2230 (36.3%) | 293 (38.1%) | 0.27 | 2481 (22.8%) | 1928 (22.7%) | 0.94 | 1285 (30.2%) | 881 (29.9%) | 0.96 |
| **Multiple pregnancy** | 53 (0.9%) | 8 (1.0%) | 0.18 | 86 (0.8%) | 85 (1.00%) | 0.12 | 105 (2.5%) | 81 (2.8%) | 0.20 |
| **GA at enrolment** | 10.4  [7.9; 14.1] | 12.4  [8.6; 17.4] | <0.001 | 18.7  [13.6; 24.6] | 22.5  [15.6; 30.4] | <0.001 | 25.1  [19.5; 30.9] | 27.9  [21.1; 34.6] | <0.001 |
| **Maternal basic education** | 3560 (57.9%) | 415 (53.9%) | 0.25 | 2486 (22.8%) | 1305 (22.8%) | <0.001 | 2486 (58.5%) | Unknown | Unknown |

*CLIP (Community Level Interventions in Pre-eclampsia), GA (gestational age), IQR (interquartile range), POM (Piers ON the Move), wks (weeks)*

** The p value was based on comparisons of women who did and did not receive POM-guided care (abbreviated as having “received POM’ in the table), for each country, by a multilevel regression model as appropriate. Data are not available for Nigeria for women who did not receive POM-guided care because of the lack of surveillance data.*
