# Supplementary material for: The incidence of pregnancy hypertension in India, Pakistan, Mozambique, and Nigeria: A prospective population-level analysis
Source: PLoS Med. 2019 Apr 12;16(4):e1002783. doi: 10.1371/journal.pmed.1002783 (PMC6461222; doi:10.1371/journal.pmed.1002783)
Supplement: S6 Table — (DOCX) [file pmed.1002783.s006.docx]

**Table S6: Sensitivity analysis including all women who had a POM-guided visit regardless of follow up status**

|  | **India** | **Pakistan** | **Mozambique** | **Nigeria** | P |
| --- | --- | --- | --- | --- | --- |
| N women | 7054 | 11399 | 4781 | 8790 |  |
| Hypertension | 665 (9.4%) | 1025 (9.0%) | 478 (10.0%) | 766 (8.7%) | 0.08 |
| **Hypertension type at diagnosis**  **of elevated blood pressure** |  |  |  |  |  |
| Chronic hypertension | 67/5411 (1.2%) | 69/4766 (1.5%) | 18/907 (2.0%) | 46/1770 (2.6%) | <0.001 |
| Gestational hypertension | 378/6139 (6.2%) | 700/11017 (6.4%) | 356/4641 (7.7%) | 498/8024 (6.2%) |  |
| Pre-eclampsia | 214/6139 (3.5%) | 252/11017 (2.3%) | 98/4641 (2.1%) | 205/8024 (2.6%) |  |
| Eclampsia | 1/6139 (0.0%) | 0/11017 (0.0%) | 0/4641 (0.0%) | 3/8024 (0.0%) |  |
| Hypertension diagnosis type not known | 7/665 (1.1%) | 4/1025 (0.4%) | 4/476 (0.8%) | 17/766 (2.2%) |  |
