# Supplementary material for: The incidence of pregnancy hypertension in India, Pakistan, Mozambique, and Nigeria: A prospective population-level analysis
Source: PLoS Med. 2019 Apr 12;16(4):e1002783. doi: 10.1371/journal.pmed.1002783 (PMC6461222; doi:10.1371/journal.pmed.1002783)
Supplement: S7 Table — (DOCX) [file pmed.1002783.s007.docx]

Table S7: Sensitivity analysis including adjustment for baseline characteristics (All numbers are adjusted Odds Ratios compared to India described as aOR[95%CI]; p value)

|  | **Pakistan** | **Mozambique** | **Nigeria** |
| --- | --- | --- | --- |
| **Hypertension overall** |  |  |  |
| Adjusted for age, and parity | 0.74 [0.66, 0.83];  p<0.001 | 0.97 [0.85, 1.11];  p= 0.671 | 0.82 [0.73,0.92];  p=0.001 |
| Adjusted for age, parity and level of education | 0.75 [0.67, 0.85]  p<0.001 | 0.97 [0.85,1.11];  p=0.663 | Not possible* |
| **Chronic hypertension** |  |  |  |
| Adjusted for age, and parity | 0.86 [0.58,1.27]  p=0.439 | 1.50 [0.82, 2.75];  p=0.187 | 1.68 [1.05, 2.70;  p=0.030 |
| Adjusted for age, parity and level of education | 0.91 [0.60,1.39];  p=0.673 | 1.55 [0.84, 2.85];  p=0.159 | Not possible* |
| **Gestational hypertension** |  |  |  |
| Adjusted for age, and parity | 0.83 [0.72, 0.95];  p=0.008 | 1.16 [0.99, 1.35];  P=0.059 | 0.90 [0.78,1.05];  p=0.173 |
| Adjusted for age, parity and level of education | 0.84 [0.73, 0.97];  p=0.016 | 1.16 [0.99,1.35];  p=0.064 | Not possible* |
| **Pre-eclampsia** |  |  |  |
| Adjusted for age, and parity | 0.49 [0.40, 0.60]  p<0.001 | 0.61 [0.50, 0.76]  p<0.001 | 0.52 [0.40, 0.67]  p<0.001 |
| Adjusted for age, parity and level of education | 0.50 [0.41, 0.63]  p<0.001 | 0.51 [0.40, 0.66]  p<0.001 | Not possible* |

**Surveillance data on level of education was not available in Nigeria*
