# Supplementary material for: The incidence of pregnancy hypertension in India, Pakistan, Mozambique, and Nigeria: A prospective population-level analysis
Source: PLoS Med. 2019 Apr 12;16(4):e1002783. doi: 10.1371/journal.pmed.1002783 (PMC6461222; doi:10.1371/journal.pmed.1002783)
Supplement: S3 Text — (DOCX) [file pmed.1002783.s010.docx]

**S3 Text: Community health workers –current scope of practice**

***India: Accredited Social Health Activists (ASHA)***

ASHAs are central to communication between the health care system and rural populations. ASHAs are local women trained to act as health educators and promoters in their communities. They receive a total of 28 days’ core training in five instalments, supplemented by ongoing on-the-job training and continuous professional development. After six months’ work in her village, an ASHA is then sensitised to issues related to human immunodeficiency virus (HIV) and sexually transmitted infections (prevention and referral), and trained in newborn care. ASHAs’ maternal-newborn tasks include motivating women to have a facility birth, bringing children to immunisation clinics, and encouraging family planning.

***Pakistan: Lady Health Worker (LHW)***

LHWs, female by definition, have a pivotal role in Pakistan’s health care system. LHWs have 15 months of training in maternal and child health. Each LHW covers a population of ≈1000-1500 individuals, and cover the following aspects of maternal-newborn care: health promotion, hospital referrals (‘advice to seek care from the nearest referral facility’), and basic neonatal care.

***Mozambique: Agentes Polivalentes Elementares (APE)***

APEs, who may be male or female, are basic multi-task health care helpers who help promote health in their communities and link them with the national health system. APEs are recruited from within their communities and undertake a four-month residential training programme which focusses on preventative medicine, and training on curative care centred on child health. For maternal health, the current APE curriculum is focussed on health promotion in pregnancy, including family planning and prevention of and screening for HIV and other sexually transmitted infections, as well the identification of emergency conditions, including seizures.

***Nigeria: Community Health Extension Worker (CHEW)***

CHEWs, who may be male or female, are responsible for community diagnosis and treatment of minor ailment and diseases; assisting mid-level health workers in providing care at health facilities; and community outreach. Their 24-36-month training relevant to CLIP covers: English language , computer skills, reproductive health (but not pregnancy hypertension or other complications), clinical skills, and referral system and outreach services. The CHEW programme includes certification in community health, practical exercises and examinations, supervised clinical and community experience. CHEWs work both in the community and clinic settings; however due to workload constraints, CHEWs often remain stationed predominantly at the PHC.
